# Evolutionary Signal and Development of the Foramen Dorsum Sellae (Nov.) Across Primates

**DOI:** 10.1002/jmor.70164

**Published:** 2026-08-26

**Authors:** Stephanie L. Canington, Zana R. Sims, Alexandria L. Sambrano, Christian A. Mastroianni, Michael L. Platt, Timothy D. Smith, Valerie B. DeLeon, Myra F. Laird

**Affiliations:** ^1^ Department of Basic & Translational Sciences University of Pennsylvania School of Dental Medicine Philadelphia Pennsylvania USA; ^2^ Department of Ecology, Evolution, & Behavior University of Minnesota Saint Paul Minnesota USA; ^3^ Department of Evolutionary Anthropology Duke University Durham North Carolina USA; ^4^ DMD Program University of Pennsylvania School of Dental Medicine Philadelphia Pennsylvania USA; ^5^ Department of Neuroscience, Perelman School of Medicine University of Pennsylvania Philadelphia Pennsylvania USA; ^6^ Department of Psychology, School of Arts and Sciences University of Pennsylvania Philadelphia Pennsylvania USA; ^7^ Marketing Department, Wharton School of Business University of Pennsylvania Philadelphia Pennsylvania USA; ^8^ Department of Health and Rehabilitation Sciences, Program of Physical Therapy Slippery Rock University Slippery Rock Pennsylvania USA; ^9^ Department of Anthropology University of Florida Gainesville Florida USA

**Keywords:** foramen, phylogeny, primates, sella turcica

## Abstract

The sella turcica, a small depression within the sphenoid bone in mammal skulls, exhibits notable morphological variation across primates, including the occasional presence of accessory openings. We investigated one such foramen within the posterior wall of the sella turcica that has been variably described in humans and other primates. We scored foramen presence in humans (*N* = 114) and 30 genera of non‐human primates (*N* = 1468), tested its phylogenetic signal, conducted human and macaque dissections, and tracked its developmental onset with newborn and infant baboons. The foramen was present in 12% of humans and 33% of non‐human primates, occurring most frequently among hylobatids (77%) and non‐human great apes (59%) and least among strepsirrhines (4%), revealing a significant phylogenetic signal (*p* < 0.001). Phylogenetic generalized least squares showed a strong signal with presence regressed on cranial base angle (CBA), a measure of cranial flexion. Genera with lower presence were more likely to have larger CBAs (Pagel's *λ* = 0.891), though this signal was strongly driven by *Homo*. Neither dissections nor developmental histology revealed foramen neurovasculature. However, one fetal and one infant baboon showed spatial constraints by the neurohypophysis, which may compress the sella and delay or prevent local endochondral ossification. We propose formalizing the *foramen dorsum sellae* and suggest its formation relates to developmental variation in dorsum sellae ossification and remodeling.

## Graphical abstract

The dorsum sellae of the sphenoid bone occasionally exhibits an accessory opening, though its significance is unknown. We scored its presence in humans (*N* = 114) and 30 non‐human primate genera (*N* = 1468) and combined histological, dissection‐based, and phylogenetic approaches to understand its function. The foramen occurred in 12% of humans and 33% of non‐human primates (primarily apes) and lacked associated neurovasculature. We propose formalizing the *foramen dorsum sellae* and suggest its formation relates to developmental variation in dorsum sellae ossification and remodeling.

## 1 Introduction

**Figure 1:**
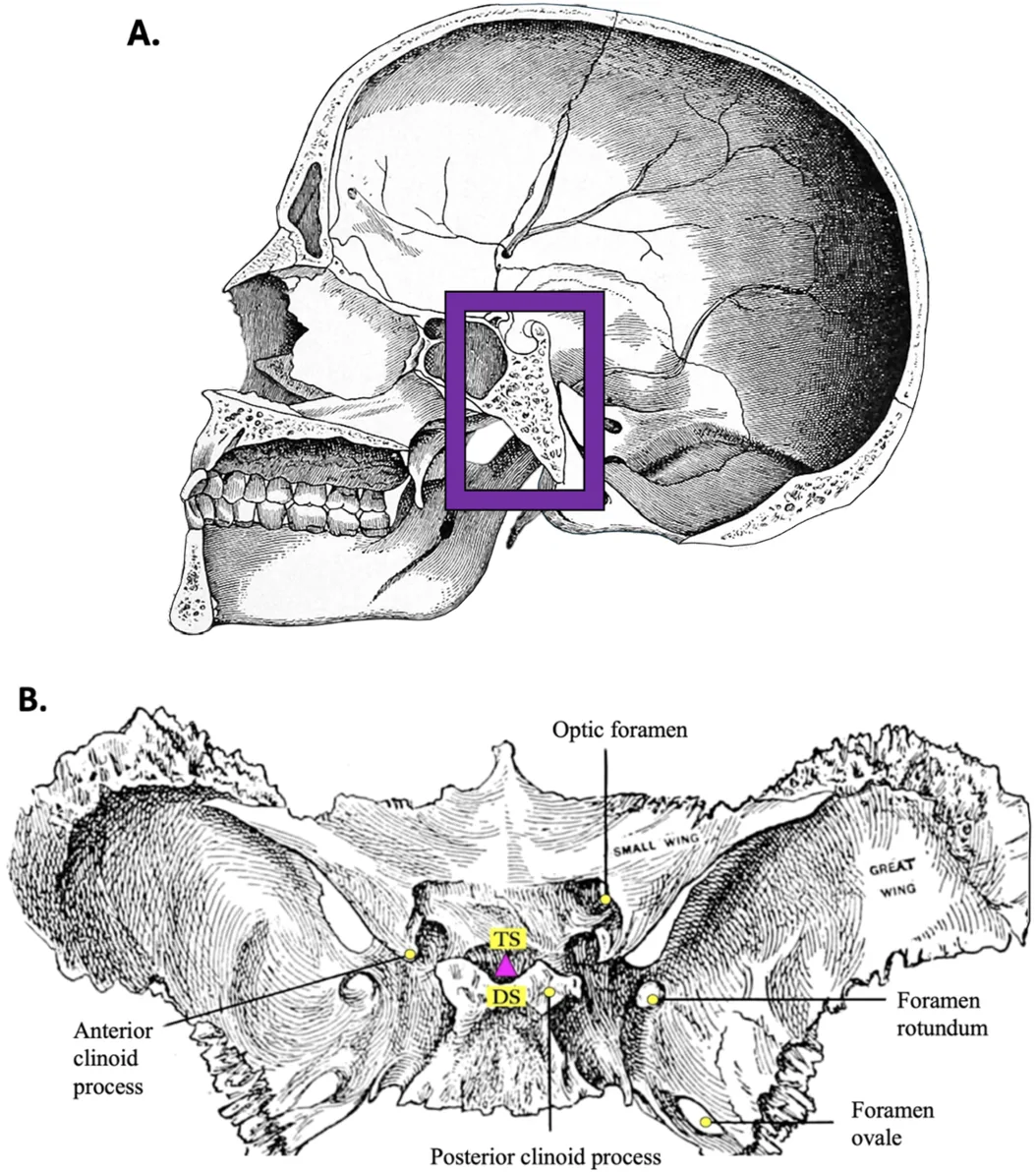
Illustration of the human sella turcica. (A) Location of the sella turcica within the cranial base, as demarcated by a purple rectangle. Modified from Anatomy, Physiology, and Hygiene for High Schools (1900; public domain). (B) Sphenoid bone of the middle cranial fossa; TS: tuberculum sellae; pink triangle: hypophyseal fossa. Modified from Piersol's Human Anatomy (1919; public domain).

At the base of the mammal skull, the middle cranial fossa is a complex region rich with cranial nerves and venous networks. Critically, it houses the pituitary gland within a depression of the sella turcica, at the center of the sphenoid bone. The sella turcica is marked anteriorly by the tuberculum sellae (TS) and posteriorly by the dorsum sellae (DS). The DS is a thin plate that is continuous with the clivus of the posterior cranial fossa, and includes two posterior clinoid processes that anchor the tentorium cerebelli (Figure 1).

The morphology of the sella turcica varies widely across mammals and within primates, reflecting differences in DS form and basicranial size, shape, and postnatal growth trajectories (Camp 1923; Hofer 1969; Novacek 1993; Ross and Ravosa 1993; Bastir et al. 2010; Tekiner et al. 2015; Haider et al. 2016; Iskra et al. 2023; Koçyiğit et al. 2024; Peralta‐Pérez and Briones‐Salas 2025). Among this variation is a midline perforation within the DS that has been occasionally documented in humans (Frassetto 1903; Le Double 1903; Laschi 1931; Pribram and du Boulay 1971; Berger et al. 1976; Bonneville and Dietemann 1981) and other primates (Cercopithecidae and Hominidae; Frassetto 1903; Hrdlička 1906). We formalize this perforation as the *foramen dorsum sellae* and explore its presence across the order Primates, its neurovascular associations, and its phylogenetic, spatial, and developmental relationships with structures of the sella turcica.

Normal anatomical variations of the primate middle cranial fossa include bony outgrowths (Skrzat et al. 2014; Lang 1977), accessory venous sinuses (Tubbs et al. 2007), and foramen absence, malformation, or asymmetry (e.g., foramen spinosum – Khan et al. 2012; Özdemir et al. 2024; foramen rotundum – Shapiro and Robinson 1967). Additional intracranial foramina are common on the clivus (Inal et al. 2015), floor of the sella turcica (Bonatto‐Costa et al. 2020), and resulting from caroticoclinoid ligament ossification (Gibelli et al. 2018). These foramina are often considered epigenetic in origin and appear with varying frequency in human populations (Hauser and De Stefano 1989). Many of these foramina are also functional, meaning that they form around a vessel or neurovascular bundle and facilitate anastomotic connections in the area. One possibility is that the foramen in the DS is similarly functional, given the ubiquity of vasculature in this area. For instance, a venous sinus is sometimes present within the DS connecting to the cavernous and petrosal sinuses and the basilar plexus (Schnitzlein et al. 1985).

### 1.1 The Primate Basicranium

Spatial arrangements between bones and the degree of skeletal plasticity during development play a role in the morphology of the cranium. The influence of these factors depends on the degree of integration across the skull (Esteve‐Altava 2022), participation of specific bones in modular units (Schuurman and Bruner 2025), and the presence of features such as large brains or eyes. In the case of brain expansion, primates exhibit different rates, onsets, and durations of growth (Lieberman, Ross, et al. 2000), with maturation of form of the facial skeleton occurring after that of the basicranum (Moore and Lavelle 1974; Bastir et al. 2006). Therefore, the formation of a foramen in the DS may reflect spatial variation in the basicranium.

Mechanical loading is a key regulatory factor of bone adaptation throughout the body (Herring and Ochareon 2005). Cranial growth specifically may be influenced by these loads, with slower rates of cartilaginous growth along the axis of compressive forces (Herring 1993). As purely intracranial structures, the growth of the bones of the post‐sellar region is affected by intracranial mechanical pressures from the brain, the dura mater, and cerebrospinal fluid (Moss and Young 1960). A combination of a flexed basicranium and a rounded braincase reduces mechanical stresses to the post‐sellar cranial base, including the clivus and dorsum sellae (Demes 1985). These changes in mechanical stresses affect longitudinal bone deposition and remodeling of the DS that may influence the patterns of foramen presence across taxonomic groups.

The positioning and morphology of the sella turcica are central to the spatial variability of the cranium. Cranial base angle (CBA) is measured as the angle connecting the sphenoid to the basicranium and anterior cranial fossa (Lieberman, Pearson, et al. 2000), and increased flexion of this angle has been proposed as a mechanism of solving intracranial space issues coinciding with increased brain size (but not an isometric increase in basicranial length, Ross and Ravosa 1993; Strait 1999). Larger CBAs result in a shift in the positioning of the tuberculum sellae relative to the DS that forms a ventrally open angle (< 180°), as opposed to the structures lying in a relatively horizontal plane (Ross and Ravosa 1993). For example, sphenoidal shortening in *Homo* has significantly reduced facial projection and increased cranial globularity through a relative decrease in cranial length, making our genus distinct from any other primate (Lieberman 1998). Tarsiers similarly possess a highly flexed basicranium (Ross and Ravosa 1993), though this likely coincides with their unique and highly modified orbital evolution (e.g., Rosenberger 2010). CBA therefore serves as an important measurement for tracking phylogenetic and evolutionary changes in relative brain and cranial size and shape across Primates, and influences the morphology of the sella turcica (Ross and Ravosa 1993; Strait 1999; Ross and Henneberg 1995). However, the association between CBA and an opening in the DS is unknown.

### 1.2 Pathological and Developmental Considerations

Pathological conditions such as osteoporosis, pituitary adenomas, elevated intracranial pressure, and arterial hypertension may result in the demineralization of the DS and floor of the sella turcica in humans (Fry and du Boulay 1965; Bonneville and Dietemann 1981; Canci et al. 1992). However, the presence of a foramen in individuals with normal skull morphology indicates that this feature is likely not the result of pathological conditions. The presence of a foramen in the DS may also result from developmental malformations. Most of the sella turcica is formed from a cartilaginous postsphenoid fusing with its anterior counterpart, the presphenoid (Bonneville and Dietemann 1981; Som and Naidich 2013). In human fetuses, the pre‐ and post‐sphenoid initiate unification along their lateral margins, forming an intrasphenoidal synchondrosis that fully ossifies prior to birth (Bonneville and Dietemann 1981; Grzonkowska et al. 2024). Fusion of the intrasphenoidal synchondrosis in humans, which occurs perinatally (Baume 1968), may also affect CBA and facial angulation in primates (Jeffery and Spoor 2002; Mano et al. 2021). At birth, the human DS is cartilaginous (Berger et al. 1976) with full ossification around 4 years (Bonneville and Dietemann 1981), although the plate itself continues to increase in height and width until puberty (Berger et al. 1976). In non‐human primates, DS ossification timing is unknown, although radiographic images of 12‐month‐old *Macaca mulatta* show what appears to be a relatively, if not fully, ossified DS (Michejda 1972). Further, Smith and colleagues (2020) report that in newborn strepsirrhines, the DS is fully ossified in *Varecia* but cartilaginous in *Eulemur* and *Galago*.

Developmental malformations could potentially result in the formation of a foramen in the region of the DS. One relevant example is the craniopharyngeal canal, a patent communication between the nasopharynx and sellar floor that is thought to represent an incomplete closure of Rathke's pouch (Abele et al. 2014). This structure is rare in adult humans (< 1% of population), adult Cercopithecidae (1%–14%), and *Varecia* (probable; Smith et al. 2020), but is more common in gibbons, chimpanzees, and orangutans (12%–74%, Schultz 1944; Hill 1966). Although this illustrates the occurrence of relevant developmental anomalies in the sellar region, none of these studies indicate the presence of a coincident foramen in the DS.

### 1.3 Study Aims

Previous literature has noted the presence of a foramen in the DS in individuals without obvious genetic or pathological conditions (Frassetto 1903; Le Double 1903; Hrdlička 1906; Berger et al. 1976; Bonneville and Dietemann 1981). Here, we formalize the term *foramen dorsum sellae* and explore the comparative biology of this feature using a series of phylogenetic, anatomical, and developmental investigations. First, we document foramen prevalence and phylogenetic distribution across Primates, including humans. Second, we test spatial relationships between foramen presence and cranial base flexion across the order. Third, we explore possible associations between the foramen and surrounding neurovasculature through dissections. Finally, we use microCT and histological approaches to investigate the possible developmental origins of the foramen. Through these methods, we test the following hypotheses:

Hypothesis 1 Functional foramen hypothesis. The presence of a foramen serves as a conduit for neurovascular structures in and around the middle cranial fossa. This will be traced phylogenetically as this feature is likely to have been evolutionarily beneficial to the particular cranial morphology present within lineages, including cranial base flexion.

Hypothesis 2 Developmental remnant hypothesis. Absent apparent function, the foramen is the remnant of incomplete ossification of the dorsum sellae during development. While this may be tied to phylogeny or endocranial morphology (e.g., ossification timings or biomechanical constraints), it may also appear across the order without a strong phylogenetic signal.

## 2 Materials and Methods

### 2.1 Sample

We include two categories of human remains: skulls curated by the University of Pennsylvania Schools of Medicine and Dental Medicine, and human donors who gave informed consent prior to death. The use of human donors for this project was reviewed and approved by the University of Pennsylvania Cadaver and Body Parts Operational Committee (#86801/01).

Legacy anatomical collections have a problematic history (Jones 2023; Cornwall et al. 2024), and we recognize that historic teaching collections were likely assembled from the remains of non‐consenting individuals.

To maximize genus‐level coverage, crania were surveyed across multiple species (Figure S1; Tables S1 and S2). The physical crania of 114 human and 1468 non‐human primates were scored for the *foramen dorsum sellae* in 10 physical collections: *Academy of Natural Sciences, Drexel University* (Philadelphia, PA); Bell Museum (Minneapolis, MN); *Field Museum of Natural History* (Chicago, IL); *Natural History Museum of Los Angeles County* (Los Angeles, CA); *National Museum of Natural History, Smithsonian Institution* (Washington, DC); *Natural History Society of Maryland* (Baltimore, MD); *New York University* (New York, NY); *Penn Museum, University of Pennsylvania* (Philadelphia, PA); *University of Pennsylvania Schools of Medicine and Dental Medicine legacy teaching collections* (Philadelphia, PA); and microCT scans from MorphoSource (www.morphosource.org; Boyer et al. 2016; Table S1). Visualization of microCT samples was performed using Dragonfly 3D World 2024.1 (Comet Technologies Canada Inc.; http://www.theobjects.com/dragonfly).

Only crania lacking obvious pathological conditions, with full adult dentition (or final tooth erupting), and with an intact DS were included. The DS were first scored as either foramen present or foramen absent. Those in the absent category were secondarily scored as either solid, bone with no impression of a foramen, or translucent, presence of a round impression in the central portion of the DS covered by a thin layer of bone. Translucent versus solid plates were not statistically compared. The sample included humans and 30 genera of non‐human primates. Sex was unavailable for all 68 human crania. For the non‐human primates, females were 39.8% and males 45.7%, with the rest as sex unknown (14.5%). Additionally, 74.1% were from wild populations, 14.3% from zoos or laboratories, 0.34% from a semi‐captive facility (Duke Lemur Center, Durham, NC), and the remaining specimens were listed as locality unknown (11.2%; Table S2).

Our sample includes all four genera of great apes (Hominidae), two genera of small apes (Hylobatidae), nine genera of catarrhine monkeys (Cercopithecidae), 12 genera of platyrrhine monkeys (Aotidae, Atelidae, Callitrichidae, Cebidae, and Pitheciidae), and four genera of strepsirrhines (Indriidae and Lemuridae). The two major clades of Primates, Haplorhini (tarsiers, monkeys, and apes) and Strepsirhini (lemurs and lorises), diverged approximately 71.3 million years ago (mya; Craig et al. 2024). The platyrrhine‐catarrhine split is dated to 42.2 mya, with apes branching off around 30 mya (Reis et al. 2018; Craig et al. 2024). The species in this study range considerably in body mass (*Saguinus*: 0.418 kg to *Gorilla*: 170.4 kg; Smith and Jungers 1997), brain size (Kay et al. 2025), and ecological and dietary niches (Chapman and Chapman 1990; Scott et al. 2018).

### 2.2 CBA Analyses

Average CBA for all 31 genera was gathered from the literature (Ross and Ravosa 1993; Strait 1999). CBA was calculated as the inferior angle between two planes – the *clivus ossis occipitalis* (a line created between the basion and the posterior edge of the spheno‐occipital synchondrosis [SOS]) and the *planum sphenoideum* (a line created between the apex above the chiasmatic sulcus and the apex of the cribriform plate; see Ross and Ravosa 1993). CBA values for all genera are available in Table S3.

### 2.3 Dissections

To explore whether the foramen relates to neurovasculature in the middle cranial fossa (e.g., cavernous sinus), we conducted dissections in 46 human donors (18 female, 28 male) and three adult macaques (*M. mulatta*; two female, one male). The macaques had been euthanized for unrelated studies (University of Pennsylvania Institutional Animal Care and Use Committee #805870). For dissections, the brain was first removed to access the middle cranial fossa. Where possible, overlying dura on the DS was removed to look for the presence of a foramen from the posterior side, and if present, any structures passing through or near the foramen. To look for the presence of a foramen and any associated structures from the anterior side, the diaphragma sellae and pituitary gland were laterally displaced within the hypophyseal fossa. The dissected DS was scored using the same criteria as the skeletal samples.

### 2.4 Developmental Sample

To assess the developmental role of the foramen, µCT scans of nine captive‐born, neonatal/postnatal baboons (*Papio* sp.) were studied to determine if, and when, a foramen may begin to form. Eight specimens were scanned at the Northeast Ohio Medical University (NEOMED) using a Scanco vivaCT 75 scanner (scan parameters: 70 kVp; 114 µA; Slippery Rock University Institutional Animal Care and Use Committee #2024‐07 T). The volumes were reconstructed using 20.5–30 µm cubic voxels and exported as 8‐bit TIFF stacks for three‐dimensional reconstructions. One fetus (Papio109) was prepared for diffusion iodine‐based contrast‐enhanced CT (diceCT) to permit visualization of soft tissues. The specimen was submerged in a series of 3.75% buffered Lugol's solutions for 8 weeks (modified from Dawood et al. 2021). The stained specimen was scanned at the University of Florida Nanoscale Research Facility using a GE v|tome|x m240 scanner (scan parameters: 160 kV; 200 µA) and reconstructed using 0.0420 mm cubic voxels.

Five specimens, including three fetuses and two neonates, were histologically sectioned in the sagittal plane (Table 1). Three were previously sectioned for a prior study (Smith et al. 2021), and two specimens were histologically prepared using the same methods. Briefly, heads were decalcified in a sodium citrate–formic acid solution and then bisected. After decalcification, one‐half was used for sagittal histology. The heads were trimmed, and the basicrania were paraffin‐embedded in blocks, divided in the body of the basisphenoid (BS), and transected. Using a Micron rotary microtome, the blocks were sectioned at 10 µm thick, and every tenth section was mounted on glass slides. Slides were alternately stained using Gomori trichrome or hematoxylin‐eosin procedures.

**Table 1:** Developmental sample of *Papio*.

Specimen ID	Histology and/or µCT	Developmental Stage
Papio 101	Both	Neonate, postnatal day 2
Papio 102	µCT	Late‐stage fetal, near term (168 days gestation)
Papio 103	µCT	Neonate, postnatal day 0
Papio 104	µCT	Neonate, postnatal day 0
Papio 106	µCT	Neonate, postnatal day 0
Papio 107	Both	Late‐stage fetal (150 days gestation)
Papio 108	µCT	~1 year infant
Papio 109	Both	Mid‐stage fetal (129 days gestation)

We supplemented our developmental sample with a collections‐based survey using the same DS scoring protocols in a sample of 22 juvenile and subadult orangutans (*Pongo*). All specimens possessed unerupted third molars (Table S4). *Pongo* was selected due to availability, and all specimens were surveyed at the National Museum of Natural History (Smithsonian Institution) and the Philadelphia Academy of Natural Sciences (Drexel University).

#### 2.4.1 Analyses

Power analyses were conducted in R (R Core Team 2025) using the package “pwr” (Champely et al. 2018) to evaluate sample size strength for each genus as it related to the relationship between foramen presence and scorable sample size (Table S5). Genera with a power of ≤ 0.5 were excluded unless the sample contained at least 30 individuals (Table S2). Genera with a low power but acceptable sample size included: *Pan* (75 scored; power = 0.052), *Callithrix* (59 scored; power = 0.400), *Papio* (71 scored; power = 0.132), and *Colobus* (39 scored; power = 0.307).

The data were tested for phylogenetic signal using the packages “phytools” (Revell 2024) and “geiger” (Harmon et al. 2007; Pennell et al. 2014). An arcsine transformation was performed on the percentages to normalize the data. Primate phylogenetic consensus chronograms were downloaded from *10ktrees.nunn‐lab.org* (Arnold et al. 2010). A phylogenetic signal was tested with 10,000 simulations using Blomberg's *K*. Using Blomberg's *K*, phylogenetic signal is expected to lie at one, and deviations represent more or less signal than would be expected under the Brownian model (Blomberg et al. 2003). Where *K* is zero, no phylogenetic signal is detected for the trait, suggesting that the trait likely evolved independent of phylogeny (Kamilar and Cooper 2013). A character map using foramen presence/absence was constructed to visualize trait prevalence across the primate tree and estimate frequencies at ancestral nodes.

To test the relationship between foramen presence and CBA, Ordinary least‐squares (OLS) regressions were performed (R base package). To evaluate the impact of phylogeny on this relationship, we used a phylogenetic generalized least squares (PGLS) analysis and a Brownian motion model (R packages “caper” – Orme et al. 2023, and “ape” – Paradis et al. 2019). Phylogenetic signal was measured as a maximum likelihood estimate of Pagel's Lambda (*λ*; Pagel 1999).

## 3 Results

### 3.1 Occurrence of a Foramen Dorsum Sellae Across Primates

**Figure 2:**
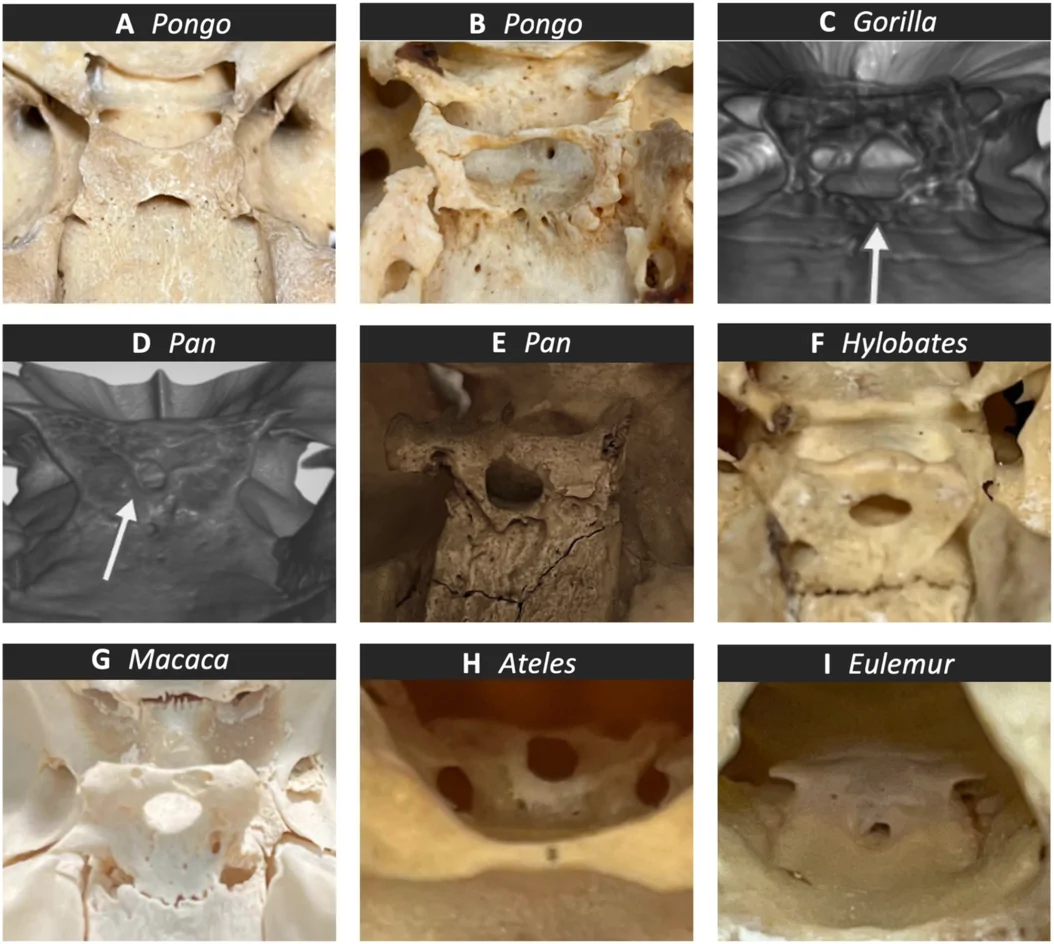
Foramen variations across non‐human primates: Hominidae – (A and B) *Pongo*, (C) *Gorilla*, and (E) *Pan*; (F) *Hylobates* (Hylobatidae); (G) *Macaca* (Cercopithecidae); (H) *Ateles* (Atelidae); and (I) *Eulemur* (Lemuridae). White arrows in (C and D) (microCT) indicate the foramen. Images are not to scale.

A total of 1582 human and non‐human primates were scored for the presence of a formaen dorsum sellae, yielding a 31.61% foramen occurrence across the order. Foramina were present in 12.28% of the human sample, 64.2% of the non‐human greater and smaller apes, 35.8% of the catarrhine monkeys, 16.6% of the platyrrhine monkeys, and 0.04% of strepsirrhines. In nearly all crania where a foramen was present, it was centrally located within the plate of the DS (Figure 2). Rarely was a foramen at or near the ossification center of the SOS (however, see Figure 2A).

Hylobatidae had the highest presence of a foramen occurring in 76.92% of the sample, including 93.75% of the *Symphalangus* specimens (Table 2). Hominidae family‐level presence was 43.8% when including *Pan, Pongo, Gorilla*, and *Homo*, and increased to 59.23% with the removal of *Homo*. The greatest presence among platyrrhine monkeys was in Callitrichidae – the family of marmosets and tamarins – at 27.97%. Of strepsirrhines, a foramen was present in only four of 96 lemur specimens – including *Varecia, Eulemur*, and *Lemur catta* (Lemuridae; 4.17%). Across the order, only two genera exhibited no foramen presence: *Lagothrix*, the woolly monkey (Platyrrhini – Atelidae; 48 scored), and *Propithecus*, the sifaka, represented by a small sample size (Strepsirrhini – Indriidae; 10 scored).

**Table 2:** Frequency of foramen presence.

Group	Family	Genus^a^	*N* scored	% Foramen
Ape	Hominidae	*Gorilla*	67	43.28%
		*Homo*	114	12.28%
		*Pan*	75	50.67%
		*Pongo*	91	78.02%
Full Hominidae summary		4 genera	347	43.80%
Nonhuman Hominidae summary		3 genera	233	59.23%
Ape	Hylobatidae	*Hylobates*	75	73.33%
		*Symphalangus*	16	93.75%
Hylobatidae summary		2 genera	91	76.92%
Platyrrhine	Aotidae	*Aotus*	42	2.38%
Aotidae summary		1 genus	42	2.38%
Platyrrhine	Atelidae	*Alouatta*	59	13.56%
		Ateles*	49	18.37%
		Lagothrix*	48	0.00%
Atelidae summary		3 genera	156	10.90%
Platyrrhine	Callitrichidae	Callithrix*	59	38.98%
		Leontopithecus*	40	20.00%
		Saguinus*	44	20.45%
Callitrichidae summary		3 genera	143	27.97%
Platyrrhine	Cebidae	Callicebus*	30	3.33%
		Cebus*	39	5.13%
		Saimiri*	32	21.88%
Cebidae summary		3 genera	101	11.88%
Platyrrhine	Pitheciidae	Chiropotes*	35	17.14%
		*Pithecia*	30	26.67%
Pitheciidae summary		2 genera	65	21.54%
Catarrhine monkey	Cercopithecidae	Cercocebus*	27	18.52%
		Cercopithecus*	64	21.88%
		*Chlorocebus*	36	83.33%
		*Colobus*	39	38.46%
		Macaca*	184	25.00%
		*Nasalis*	35	31.43%
		Papio*	71	45.07%
		*Presbytis*	31	70.97%
		Trachypithecus*	44	34.09%
Cercopithecidae summary		9 genera	531	35.78%
Strepsirrhine	Indriidae	*Propithecus*	10	0.00%
Indriidae summary		1 genus	10	0.00%
Strepsirrhine	Lemuridae	*Eulemur*	43	4.65
		*Lemur*	30	3.33%
		*Varecia*	23	4.35%
Lemuridae summary		3 genera	96	4.17%

^a^ The “*” denotes that an individual(s) was scored with a whole plate though presented with a translucent, thin sheet of bone (Figure S1).

### 3.2 Phylogenetic Analyses

**Figure 3:**
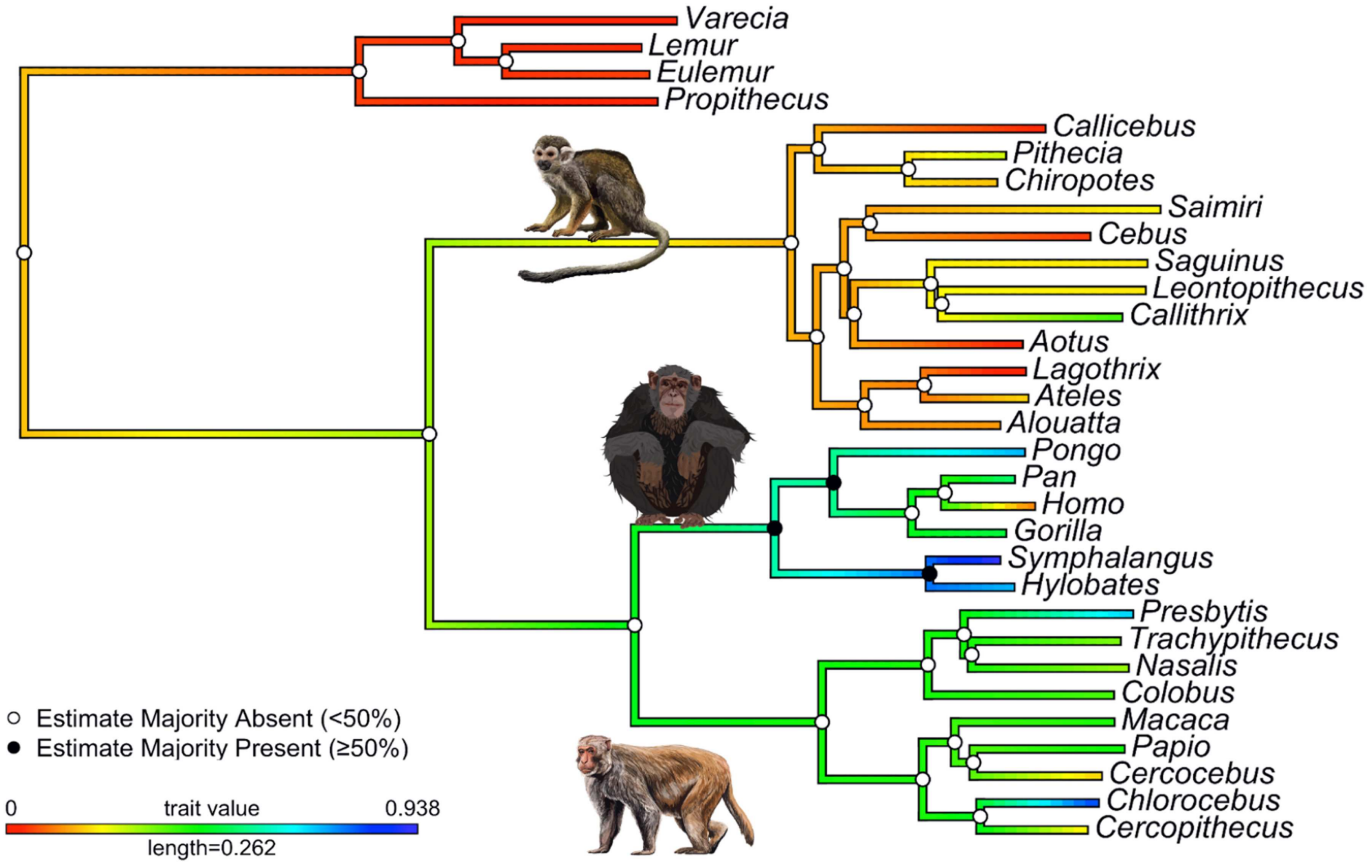
Character heat map representing foramen presence across the order Primates. Warm colors represent absence (red) to low (yellow) occurrence, while cool colors represent moderate (green) to high (dark blue) occurrence. Circles at nodes represent the estimated frequency in ancestral populations, with black representing greater than or equal to 50% and white representing less than 50% presence. Values were calculated using observed percentages for each taxa rounded to the nearest whole. Primate images from the DataBase Center for Life Science.

The presence of a foramen demonstrated a significant phylogenetic signal (*K* = 0.69, *p* < 0.001; based on 10,000 randomizations; Figure 3), suggesting that foramen presence is not a random occurrence across Primates. That said, *K* is < 1, suggesting that this trait may be more random than what can be expected under Brownian motion (Blomberg et al. 2003).

There was a significant relationship between CBA and foramen presence across all genera (*β* = −0.006539; *R* ^2^ = 0.1035; *F* = 4.465; *p* = 0.043), which became stronger with the removal of *Homo* (*β* = −0.014354; *R* ^2^ = 0.341; *F* = 16.01; *p* < 0.001; Table 3). Larger CBAs were associated with a lower foramen frequency. PGLS results show a strong phylogenetic signal when the percentage foramen occurrence is regressed on CBA. A larger CBA is more likely in taxa with lower foramen presence (Pagel's *λ* = 0.891). This signal is significantly reduced with the removal of *Homo* (Pagel's *λ* = 0.315; Table 4).

**Table 3:** OLS output for the relationship between % foramen occurrence and CBA – Excludes *Homo*.

Analysis	Parameter	Estimate	SE	*t*‐value	*p* value	Residual SE	Multiple *R* ^2^	Adjusted *R* ^2^	*F*‐statistic	*p* value
All primates	Intercept	1.3752	0.5120	2.686	0.0118					
	CBA	−0.0065	0.0031	−2.113	0.0433					
	Model fit					0.2505	0.1334	0.1035	4.465(1, 29 df)	0.0433
Excluding *Homo*	Intercept	2.6942	0.5988	4.499	0.0001					
	CBA	−0.0144	0.0036	−4.001	0.0004					
	Model fit					0.2168	0.3638	0.341	16.01(1, 28 df)	0.0004

**Table 4:** PGLS output for the relationship between % foramen occurrence and CBA.

PGLS analysis	Branch length transformations	Adjusted *R* ^2^	*F*‐statistic	DF	*p* value
Percentage foramen occurrence and CBA – All primate genera	Lambda [LM]: 0.891	0.06887	2.405	1, 18	0.1383
	Lower bound: 0.000, *p* = 0.05099				
	Upper bound: 1.000, *p* = 0.72642				
Percentage foramen occurrence and CBA – Excluding *Homo*	Lambda [LM]: 0.315	0.09894	2.976	1, 17	0.1026
	Lower bound: 0.000, *p* = 0.53901				
	Upper bound: 1.000, *p* = 0.20705				

### 3.3 Dissections

A foramen was present in 11 of the 46 dissected humans and two of the three dissected macaques. None of those with a foramen contained evidence of neurovascular structures. In one macaque, the foramen was covered by a thin layer of cartilage that was easily penetrated by the blunt probe. We also dissected the vessels that extended from the basilar plexus anteriorly, but none extended toward the central plate of the DS.

### 3.4 Developmental Exploration

**Figure 4:**
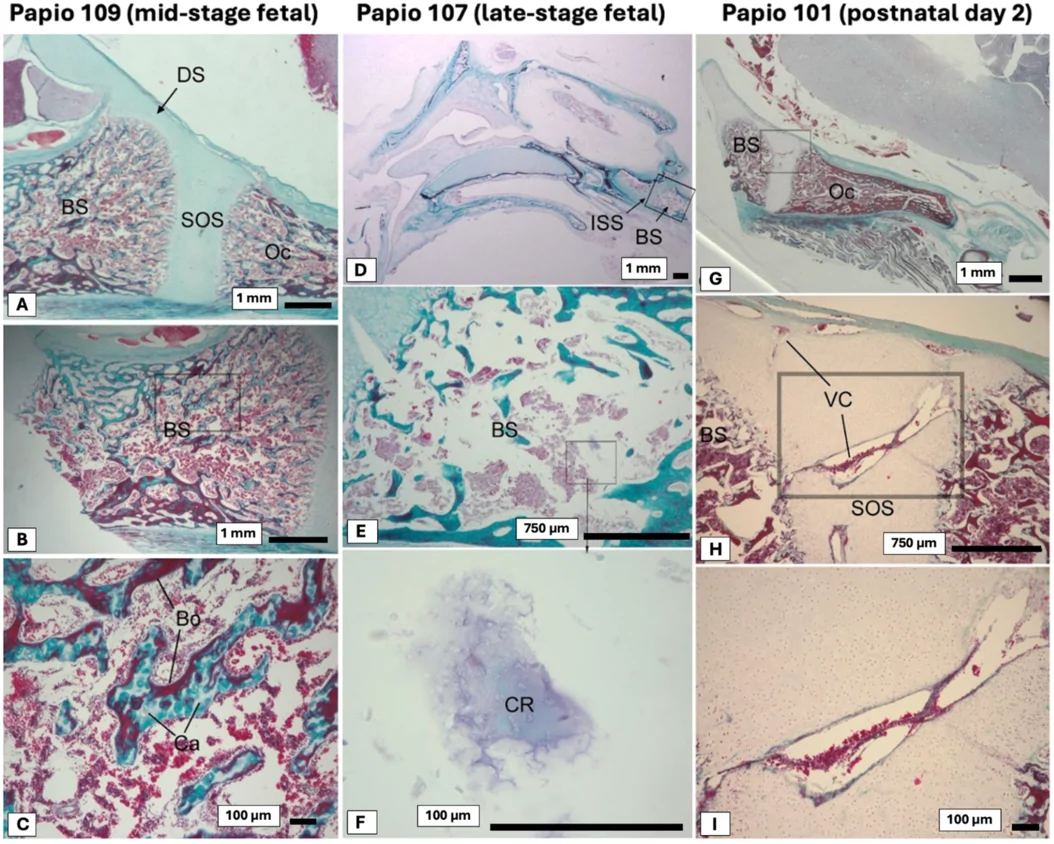
Histology: (A–C) Papio109 (mid‐fetal); (D–F) Papio107 (late fetal); (G–I) Papio101 (neonate). Note the cartilage of the rounded dorsal surface of the DS (A). From mid‐fetal (A–C) to neonatal (G–I) ages, the partially ossified DS is overlaid by cartilage matrix (light blue tissue). During mid‐fetal stages, the basisphenoid (“BS”) is most ossified ventrally (A); posterodorsally, it retains cartilage (“Ca”) matrix that is only beginning to undergo endochondral ossification (see (C); “Bo”: bone forming along a cartilage matrix). At late fetal stages, remnants of cartilage matrix are still found in the BS dorsoposteriorly (E and F). The DS and dorsal surface of the BS remain cartilaginous at birth (G), but the cartilage of the SOS and the DS region is penetrated by vascular channels (“VC”: H and I). Scale bars: (A, B, D, G) = 1 mm; (C, F, I) = 100 µm; (E and H) = 750 µm.

**Figure 5:**
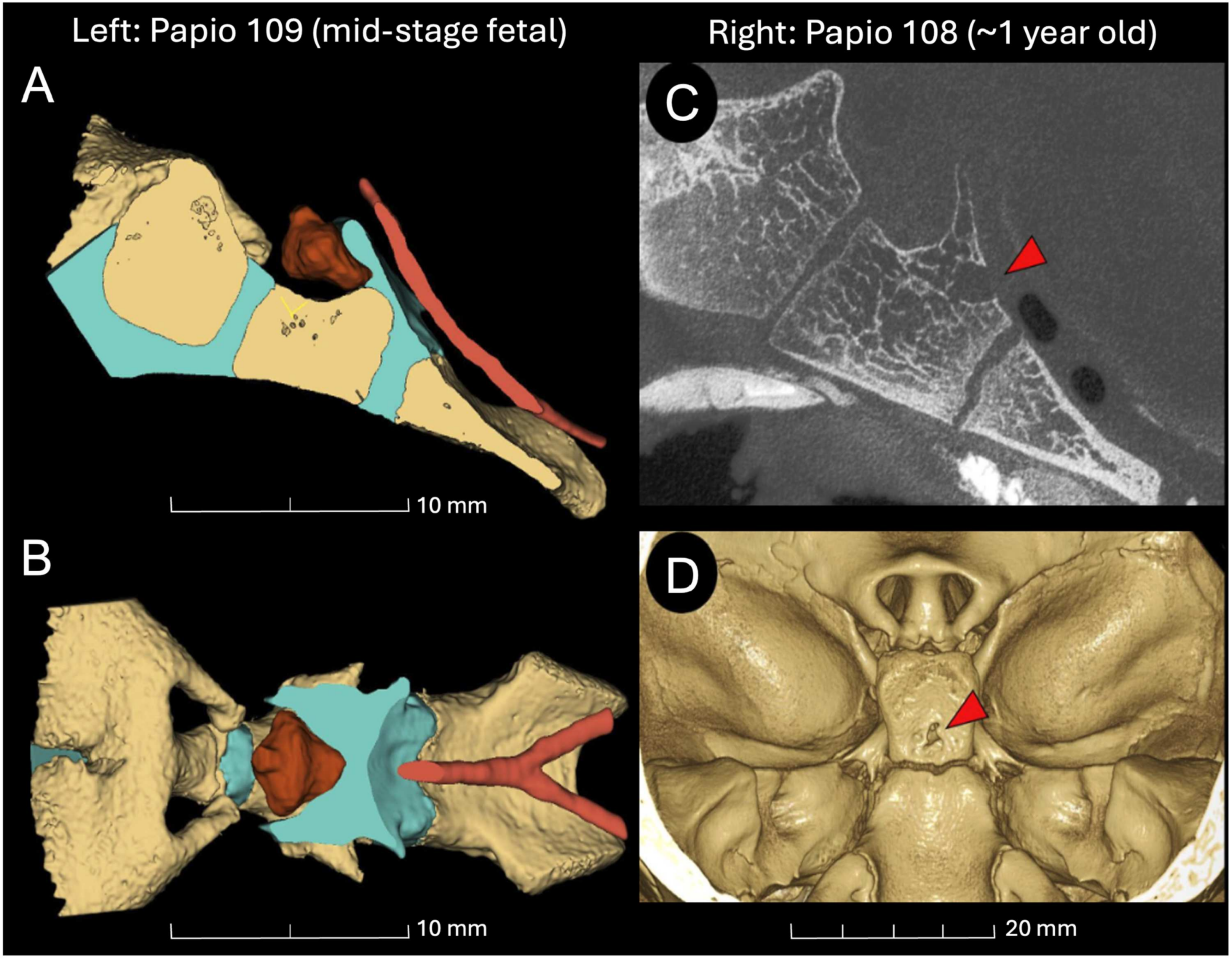
Papio109 (A and B) and Papio108 (C and D) demonstrate anterior versus posterior surface disruptions in the cartilage/laydown of bone of the DS. (A) is a 3D reconstruction of the same tissue shown in histology (Figure 4A, above). (C) is not to scale, but is a lateral view of (D).

We found preliminary evidence suggestive of foramen development in our fetal and neonatal sample of baboons (Table 1), though the dorsal aspect of the DS remains cartilaginous across fetal and perinatal ages (Figure 4A,G). The rounded dorsal surface of the DS (Figure 4A) reflects its unossified state. During mid‐fetal stages, the BS is ossified ventrally (Figure 4B), and posterodorsally it retains a cartilage matrix that is only beginning to undergo endochondral ossification (Figure 4C). Even at late fetal stages, remnants of a cartilage matrix can be found in the BS dorsoposteriorly (Figure 4E,F). The dorsal surface of the BS remains cartilaginous at birth (Figure 4G), but vascular channels penetrate the cartilage between the SOS and dorsum sellae (Figure 4H,I). In the 1‐year‐old *Papio*, the DS is mostly ossified, except for the centralized incomplete foramen (Figure 5B).

In the collections‐based survey of developmental orangutans, the DS of 11 of these individuals was incomplete or not yet formed and not scored. However, of the remaining 11 for whom the DS was complete, a foramen was present in 10 and absent in the eleventh (Figure S2). The youngest individuals with a foramen present were approximately 5 years (M^1^ erupted and M^2^ unerupted; Fooden and Izor 1983; Smith 2016).

## 4 Discussion

The middle cranial fossa is an anatomical area of clinical importance, housing the pituitary gland, cranial nerves, and rich venous plexuses. Here, we formalize the term *foramen dorsum sellae* for midline openings through the DS, and we explore the variation of this feature across Primates. While our study yielded relatively high foramen occurrence rates in the order Primates, we acknowledge that low or no occurrences in some taxa may be a product of low sample sizes, despite high statistical power. For example, our *Propithecus* sample is limited to only 10 individuals, none of whom had a foramen. Our non‐human primate dissection sample was limited to three macaques, and additional dissections, especially of non‐catarrhines, may provide additional insight into whether this foramen relates to the surrounding neurovasculature. Finally, we acknowledge that the developmental data are derived from only two taxa – one catarrhine monkey and one ape, likely poorly reflecting developmental patterns across the order. Nevertheless, both developmental approaches represent important strategies for future investigations into the formation onset and prevalence throughout ontogeny.

### 4.1 The Foramen Has Few Functional and Phylogenetic Correlates

*Hypothesis 1: Functional foramen hypothesis.* We found no evidence that the foramen is associated with the passageway of neurovascular structures in dissections or within our developmental sample. Further, there was no evidence of the basilar plexus extending towards the central DS. While our dissection sample was limited, this provides strong evidence that the foramen forms independently from the nearby neurovasculature of the cavernous sinus and basilar plexus.

Another possibility was that foramen presence is related to spatial arrangements within the cranium. Given the role of the sella turcica in determining CBA, changes to this angle may alter the attachment locations and tension of the dura in the region of the sella turcica and affect ossification of the DS (Skrzat et al. 2006; Salma et al. 2011; Rai et al. 2023). We found that taxa with a larger CBA tended to have lower foramen presence, and when including *Homo*, there was a strong phylogenetic signal between CBA and foramen presence. However, this signal is significantly reduced when *Homo* is removed. This difference likely reflects the uniquely large basicranial flexion in *Homo*, which has been related to a suite of changes in brain size, sphenoid size, orbit orientation, and basicranial length (Ross and Henneberg 1995; Strait 1999; McCarthy 2001). Further evidence of the lack of strong association between CBA and foramen presence comes from siamangs (*Symphalangus*) and vervets (*Chlorocebus*). These taxa had the highest foramen occurrence (93.75% and 83.33% of samples) and have similar CBA of 167 and 171, respectively (Table S3). *Symphalangus* and *Chlorocebus* separated approximately 30 mya (Catarrhini split; Reis et al. 2018), and it seems unlikely that phylogenetic relationships explain such high occurrences in these genera. These results suggest that spatial arrangements within the cranium play a minimal role in determining foramen presence.

The lowest rates of foramen occurrence were within Strepsirrhini, whereas the highest rates of occurrence were in hominoids. However, the *K* statistic of < 1 suggests that foramen presence or absence is not strongly conserved and that other factors contribute to both its presence and distribution. This may be demonstrated within Hominidae, where most taxa had a high rate of foramen presence (*Pongo*, *Pan*, and *Gorilla*: > 49% presence), but *Homo* had a lower rate (12.28%). In addition, Hominidae is the only group that was estimated to have greater than or equal to 50% foramen presence at ancestral nodes (i.e., the hypothetical most recent common ancestor for descendant lineages).

### 4.2 Multiple Developmental Routes to Foramen Formation

*Hypothesis 2: Developmental remnant hypothesis.* Our data suggest the *foramen dorsum sellae* arises through one or more of the following processes: (1) a disruption in the anterior cartilage of the DS in utero resulting from encroachment of the neurohypophysis (Figure 5A), (2) absent soft tissue encroachment, other localized failure of endochondral ossification, or (3) a premature cessation of ossification of the plate that may result in a cartilaginous remnant (Figure 5B). While some sphenoid foramina have no singular apparent cause (e.g., Hauser and De Stefano 1989; Abd Latiff et al. 2009; Arora et al. 2011), others are proposed to be influenced by multiple processes across different stages of development (McGonnell and Akbareian 2019). Accessory foramina in the sphenoid bone have previously been documented by other anatomical studies with no apparent singular cause (e.g., Abd Latiff et al. 2009; Arora et al. 2011).

Regardless of origin, the *foramen dorsum sellae*, as identified in our study, has remained open throughout the life of these animals. In foramina where neurovasculature is present, maintenance occurs through osteoclastic or chondroclastic activity, and varying degrees of this maintenance may affect foramen appearance across time (Akbareian et al. 2015). The lack of associated neurovasculature, along with varying degrees of patency (i.e., translucency), suggests that there are likely multiple processes involved in both the origin and variable maintenance of the *foramen dorsum sellae* in our sample.

It may be the case that impingement in the midline of the DS (e.g., caudal tail of neurohypophysis, pons, and/or basilar artery) pinches the intervening cartilage. That compression may maintain the presence of cartilage, preventing bone laydown, though future developmental work is needed to confirm this. A one‐year‐old baboon with a posterior indication of a foramen has an irregular bony margin of the DS (Figure 5B), which reinforces our interpretation of the presence of a cartilage plug contributing to the foramen as opposed to a remnant of a neurovascular channel. Our targeted developmental survey also indicates bony foramen presence in orangutans as young as five years. While our developmental baboon sample and orangutan survey do not differentiate between these proposed formation processes, these data indicate that there may be multiple pathways to foramen formation. Future study on the ossification timing in this area, the influence of adjacent soft tissue structures, and the foramen bony appearance in a range of taxa can clarify these suggestions.

It is important to note that growth rates are variable across the order Primates (Case 1978; Leigh and Terranova 1998), reflecting body size and both evolutionary and ecological constraints. The brain itself regulates the growth of the basicranium and calvarium (Braga et al. 2025), with significant variation in brain growth timing and duration (Lieberman, Pearson, et al. 2000; Leigh 2004). At present, we lack evidence that neural and/or basicranial growth rate itself is a driving factor in the occurrence of the *foramen dorsum sellae*, given its variability within families. However, future work may incorporate various aspects of cranial ontogeny, including the rates and degrees of changes in internal flexion angle (May and Sheffer 1999). As such, phylogenetic differences in foramen presence may reflect a combination of spatial and developmental factors.

Finally, we note correspondence in the presence of the *foramen dorsum* sellae and previous reports of a persistent craniopharyngeal canal. The craniopharyngeal canal has been reported as infrequent in ruffed lemurs (*Varecia)* and humans, and both taxa had low foramen DS occurrences in our survey. Similarly, the craniopharyngeal canal is reported at much higher rates among remaining Hominoidea, corresponding with 50.67%–93.75% foramen DS occurrence (Schultz 1944; Hill 1966; Smith et al. 2020). The craniopharyngeal canal is located between the nasopharynx and sellar floor, and is thought to represent an incomplete closure of Rathke's pouch (Abele et al. 2014). The relationship between these structures may indicate formation through similar patterns of developmental processes or aberrations among these taxa.

## Author Contributions

**Stephanie L. Canington:** conceptualization (equal), data curation (equal), formal analysis (equal), investigation (equal), methodology (equal), project administration (equal), resources (equal), software (equal), validation (equal), visualization (equal), writing – original draft (equal), writing – review and editing (equal). **Zana R. Sims:** data curation (equal), formal analysis (equal), methodology (equal), software (equal), validation (equal), visualization (equal), writing – original draft (equal), writing – review and editing (equal). **Alexandria L. Sambrano:** methodology (equal), writing – review and editing (equal). **Christian A. Mastroianni:** methodology (equal), writing – review and editing (equal). **Michael L. Platt:** data curation (equal), funding (equal), project administration (equal), resources (equal), writing – review and editing (equal). **Timothy D. Smith:** data curation (equal), investigation (equal), funding (equal), methodology (equal), project administration (equal), resources (equal), software (equal), validation (equal), visualization (equal), writing – original draft (equal), writing – review and editing (equal). **Valerie B. DeLeon:** data curation (equal), investigation (equal), funding (equal), methodology (equal), project administration (equal), resources (equal), software (equal), validation (equal), visualization (equal), writing – original draft (equal), writing – review and editing (equal). **Myra F. Laird:** conceptualization (equal), data curation (equal), formal analysis (equal), investigation (equal), methodology (equal), project administration (equal), resources (equal), software (equal), supervision (lead), validation (equal), visualization (equal), writing – original draft (equal), writing – review and editing (equal).

## Ethics Statement

All human donors gave informed consent for their bodies to be used for education and research prior to their death, and the use of human donors for this project was reviewed and approved by the University of Pennsylvania Cadaver and Body Parts Operational Committee (#86801/01). Use of non‐human cadaveric primate tissues was reviewed and approved by the IACUC committee at Slippery Rock University (Protocol # 2024‐07T).

## Conflicts of Interest

The authors declare no conflicts of interest.

## Supporting information


Supporting File

## Data Availability

The data that support the findings of this study are openly available in DRYAD at https://doi.org/10.5061/dryad.xsj3tx9vw. Original data for this study are available on Dryad, Supplementary Online Material, and by reasonable request to the corresponding author.
