# Supplementary material for: Evolutionary Signal and Development of the Foramen Dorsum Sellae (Nov.) Across Primates
Source: J Morphol. 2026 Aug 26;287(9):e70164. doi: 10.1002/jmor.70164 (PMC13508715; doi:10.1002/jmor.70164)
Supplement: Supplementary file 1 [file JMOR-287-e70164-s001.pdf]

A.

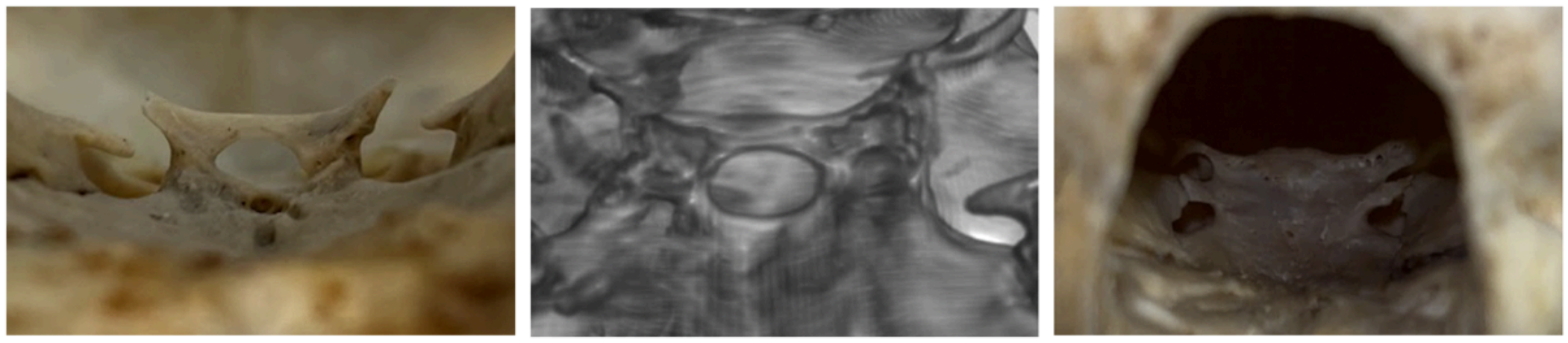

Gorilla FMNH 99092: foramen

Gorilla ISEM AGH:16: foramen

Pan USNM 220326: whole plate

B.

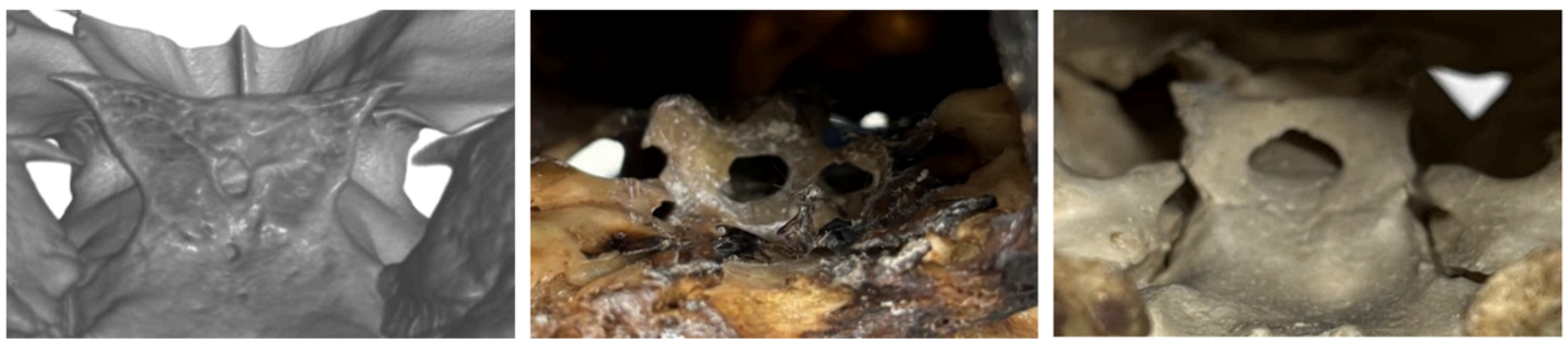

Pan MCZ 23167: foramen

Pongo USNM 142190: foramen

Pongo ANSP 22623: foramen

C.

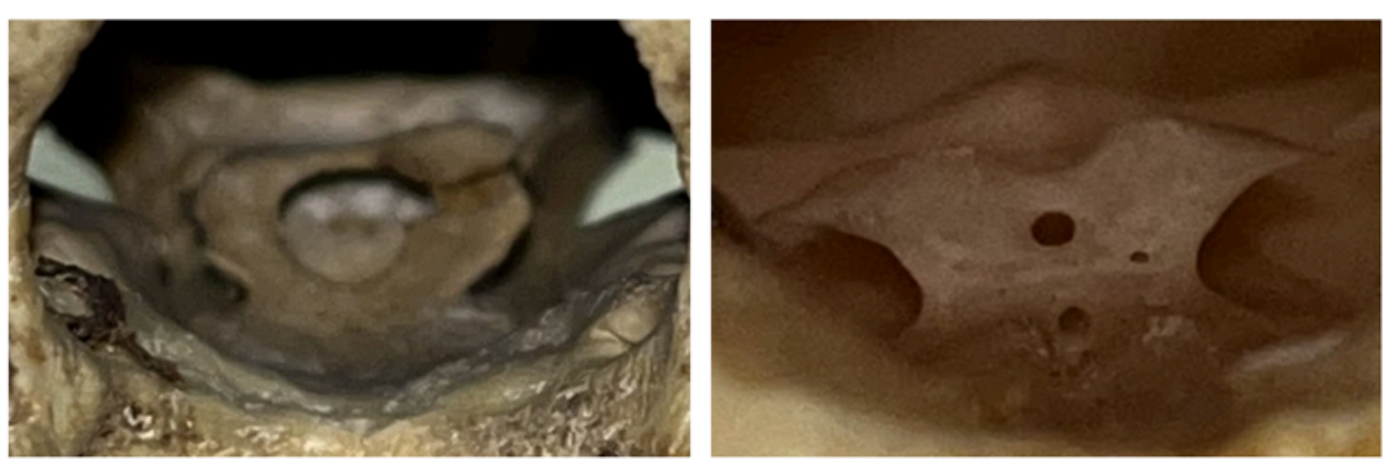

Hylobates ANSP 22623: foramen

Symphalangus ANSP 20485: foramen

D.

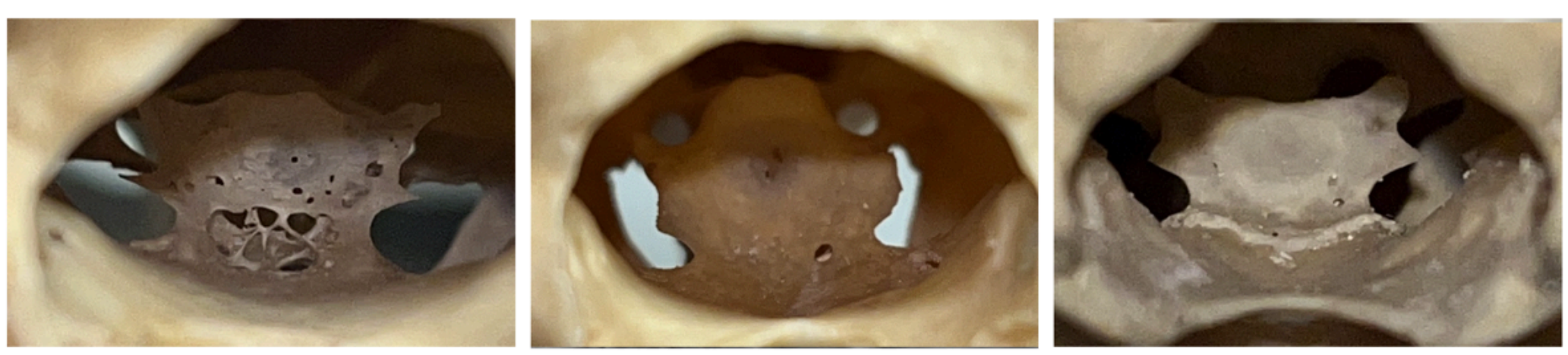

Cercocebus USNM 598488: Unknown

Cercocebus USNM 477291: translucent

Cercopithecus ANSP 12446: translucent

E.

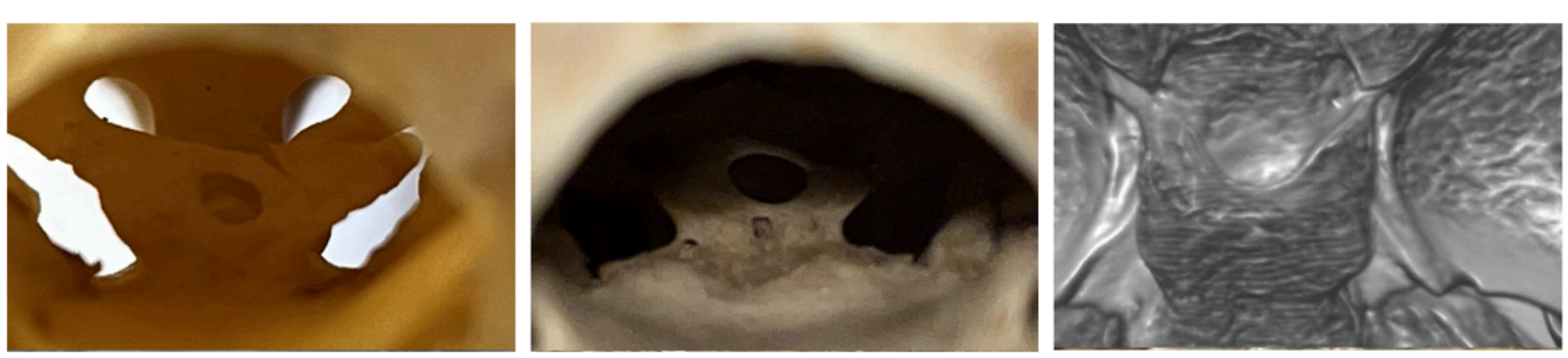

Chlorocebus FMNH 93631: foramen

Colobus USNM 481791: foramen

Colobus MCZ 47007: whole plate

F.

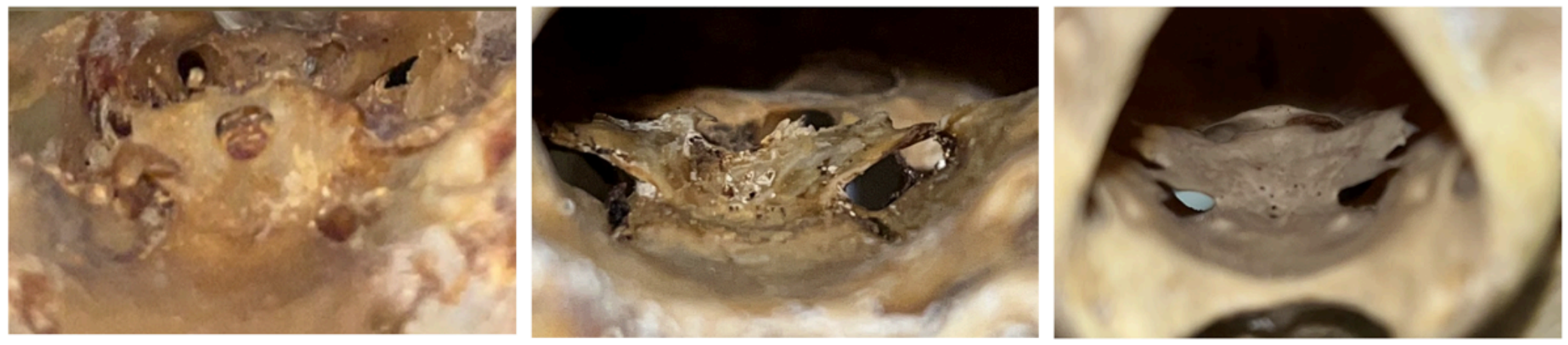

Nasalis ANSP 13994: foramen

Nasalis USNM 196786: whole plate

Papio ANSP 11833: whole plate

G.

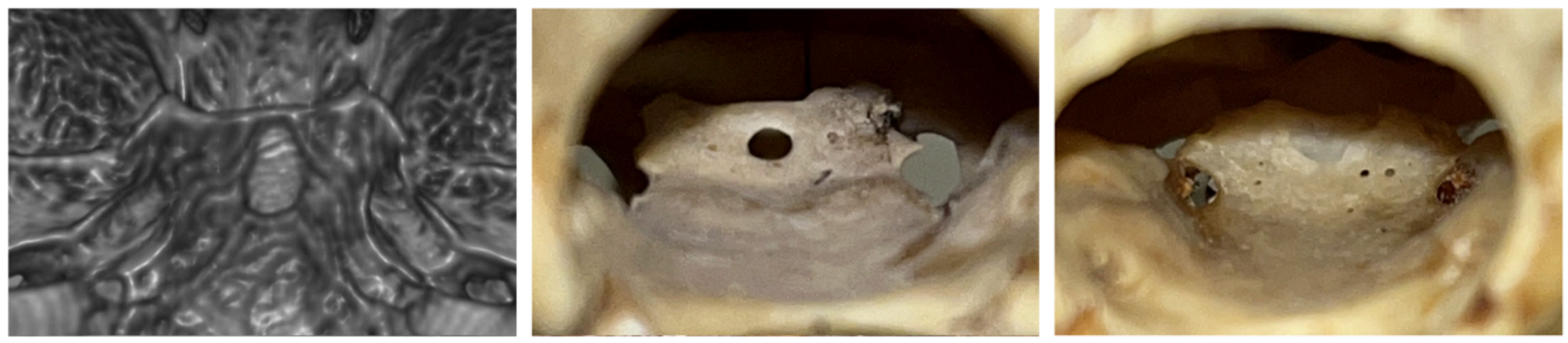

Papio SNRPC 8827 .W730: foramen

Trachypithecus USNM 251666: foramen

Trachypithecus USNM 104443: translucent

H.

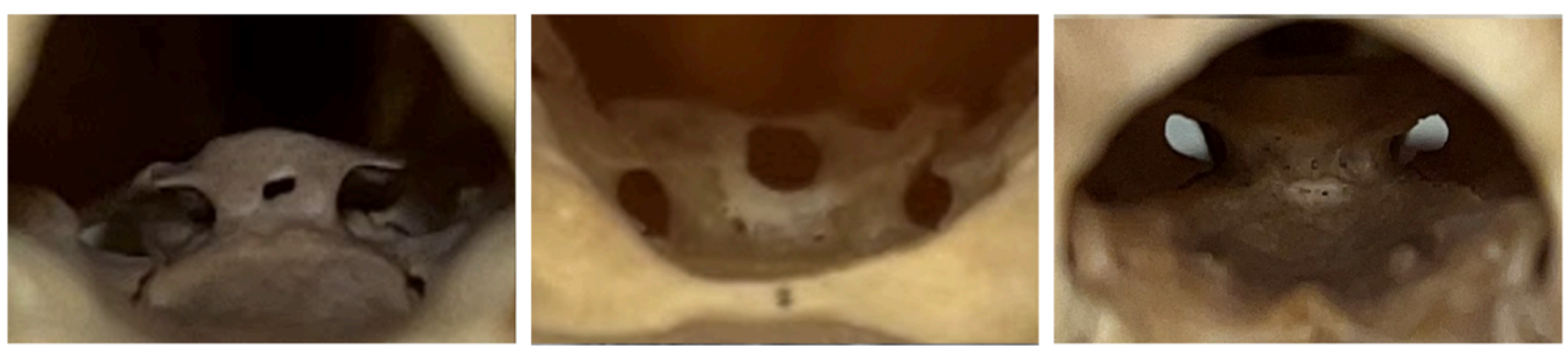

Alouatta ANSP 4792: foramen

Ateles FMNH 79943: foramen

Ateles ANSP 18859: whole plate

I.

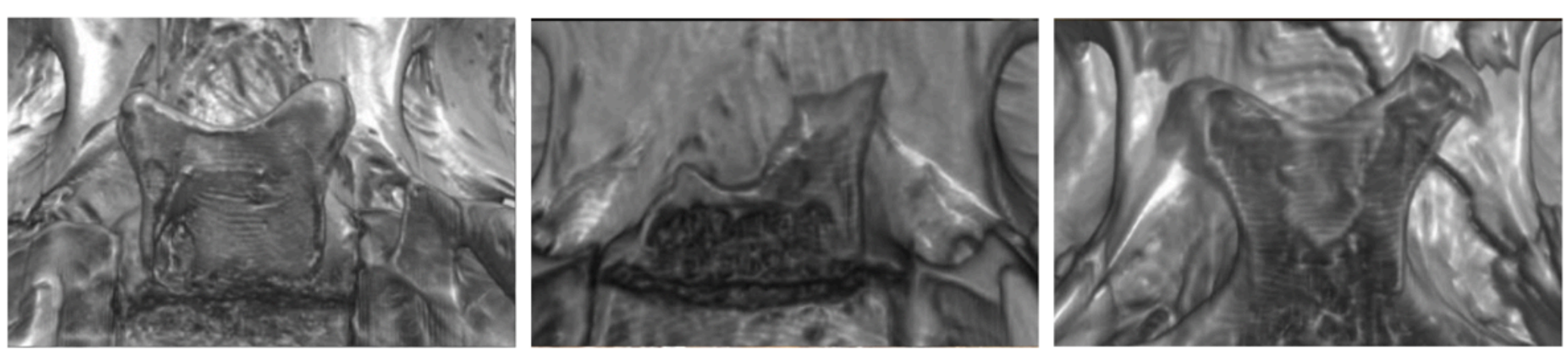

Lagothrix USNM 545879: whole plate

Lagothrix USNM 461930: unknown

Lagothrix USNM 545886: whole plate

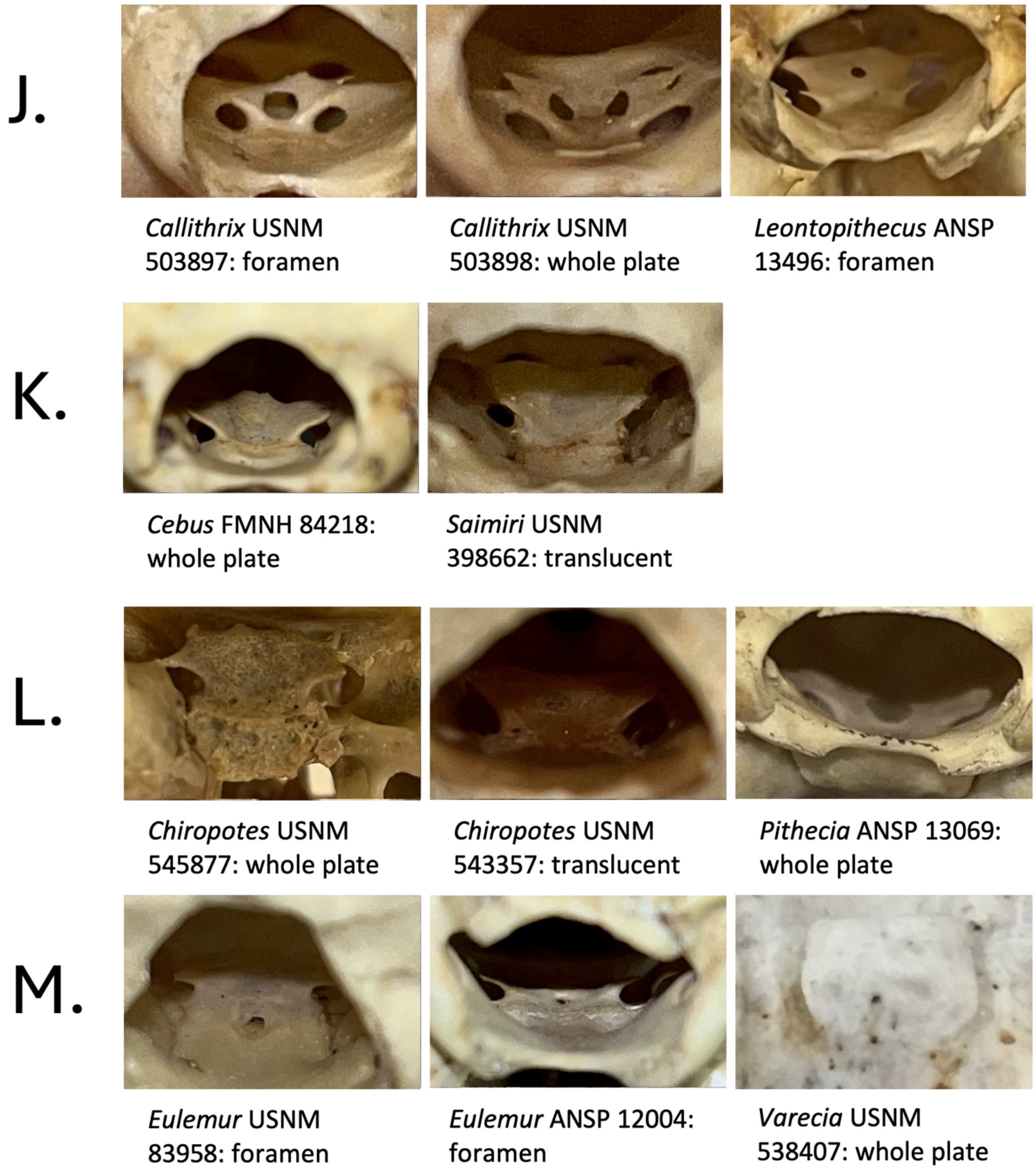

SOM Figure 1. Examples of scored non-human primate specimens. **A-B:** Hominidae; **C:** Hylobatidae; **D-G:** Cercopithecidae; **H-I:** Atelidae; **J:** Callitrichidae; **K:** Cebidae; **L:** Pitheciidae; **M:** Lemuridae. *Note*, ‘translucent’ specimens are scored as ‘whole plate’. Specimens that could not be reliably scored were labeled ‘unknown’ and excluded from all analyses (not included in sample size).

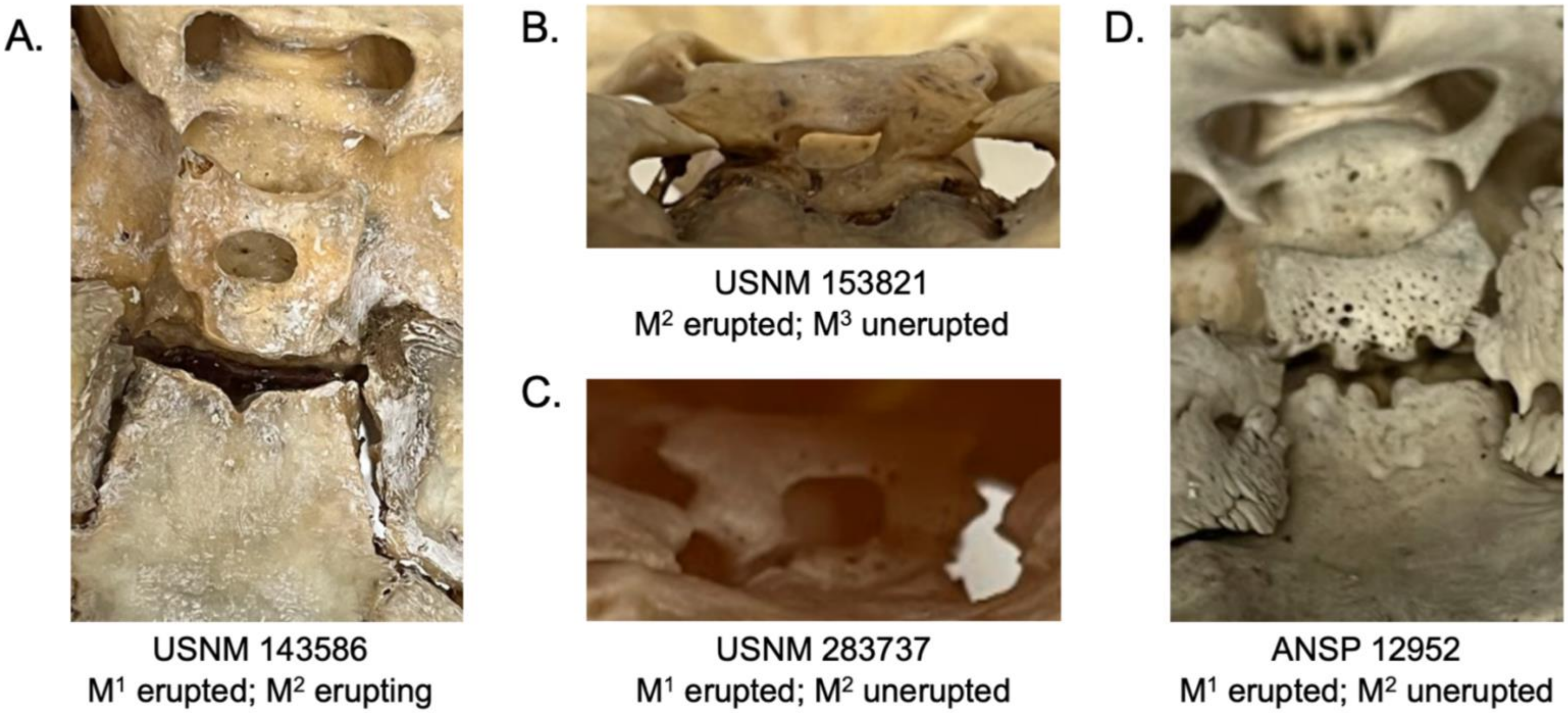

SOM Figure 2. Juvenile orangutan sample from targeted survey. Specimens (**A-C)** were all scored as possessing a foramen and specimen (**D)** was not scored as the dorsum sellae was incomplete. Note the presence of the foramen well above the spheno-occipital synchondrosis in (**A**). Images are not to scale.

SOM Table 1. Morphosource specimens (all surveyed).

Type[1]	Genus	Specimen number	Organization; Data Manager
PRIM	*Gorilla*	amnh:anth:L/ 4	AMNH Anthropology Non-Human Primates & Casts; Christopher Smith
PRIM	*Gorilla*	AMNH 202932	AMNH Mammal Collections; AMNH Mammalogy
PRIM	*Gorilla*	isem:AGH_04	Institut des Sciences de l'Evolution de Montpellier; Renaud Lebrun
PRIM	*Gorilla*	isem:AGH_15	Institut des Sciences de l'Evolution de Montpellier; Renaud Lebrun
PRIM	*Gorilla*	isem:AGH_16	Institut des Sciences de l'Evolution de Montpellier; Renaud Lebrun
PRIM	*Gorilla*	isem:AGH_17	Institut des Sciences de l'Evolution de Montpellier; Renaud Lebrun
PRIM	*Gorilla*	isem:AGH_19	Institut des Sciences de l'Evolution de Montpellier; Renaud Lebrun
PRIM	*Gorilla*	isem:AGH_21	Institut des Sciences de l'Evolution de Montpellier; Renaud Lebrun
PRIM	*Gorilla*	isem:AGH_22	Institut des Sciences de l'Evolution de Montpellier; Renaud Lebrun
PRIM	*Gorilla*	isem:AGH_23	Institut des Sciences de l'Evolution de Montpellier; Renaud Lebrun
PRIM	*Gorilla*	MCZ:Mamm:14750	MCZ Mammalogy; Harvard MCZ
PRIM	*Gorilla*	MCZ:Mamm:26850	MCZ Mammalogy; Harvard MCZ
PRIM	*Gorilla*	MCZ:Mamm:38326	MCZ Mammalogy; Harvard MCZ
PRIM	*Gorilla*	MCZ:Mamm:46325	MCZ Mammalogy; Harvard MCZ
PRIM	*Gorilla*	ucla:1788	UCLA Donald R. Dickey Bird and Mammal Collection; Deborah Bird
PRIM	*Pan*	AMNH:Mammals:M-167344	AMNH Mammal Collections; AMNH Mammalogy
PRIM	*Pan*	AMNH:Mammals:M-86857	AMNH Mammal Collections; AMNH Mammalogy
PRIM	*Pan*	MCZ:Mamm:15312	MCZ Mammalogy; Harvard MCZ
PRIM	*Pan*	MCZ:Mamm:17702	MCZ Mammalogy; Harvard MCZ
PRIM	*Pan*	MCZ:Mamm:23167	MCZ Mammalogy; Harvard MCZ
PRIM	*Pan*	MCZ:Mamm:26847	MCZ Mammalogy; Harvard MCZ
PRIM	*Pan*	MCZ:Mamm:26849	MCZ Mammalogy; Harvard MCZ
PRIM	*Pan*	MCZ:Mamm:38018	MCZ Mammalogy; Harvard MCZ
PRIM	*Pan*	MCZ:Mamm:38019	MCZ Mammalogy; Harvard MCZ
PRIM	*Pan*	MCZ:Mamm:38020	MCZ Mammalogy; Harvard MCZ
PRIM	*Pan*	MCZ:Mamm:46416	MCZ Mammalogy; Harvard MCZ
PRIM	*Pan*	MCZ:Mamm:BOM-6244	MCZ Mammalogy; Harvard MCZ
PRIM	*Pan*	sbu:87-1	Anatomical Sciences Collection; Stephanie Maiolino

PRIM	*Pan*	sbu:rs:94-1	Anatomical Sciences Collection; Stephanie Maiolino
PRIM	*Pan*	sbu:rs:94-4	Anatomical Sciences Collection; Stephanie Maiolino
PRIM	*Chlorocebus*	AMNH:Mammals:M-70063	AMNH Mammal Collections; AMNH Mammalogy
PRIM	*Colobus*	MCZ:Mamm:21147	MCZ Mammalogy; Harvard MCZ
PRIM	*Colobus*	MCZ:Mamm:22356	MCZ Mammalogy; Harvard MCZ
PRIM	*Colobus*	MCZ:Mamm:22624	MCZ Mammalogy; Harvard MCZ
PRIM	*Colobus*	MCZ:Mamm:22626	MCZ Mammalogy; Harvard MCZ
PRIM	*Colobus*	MCZ:Mamm:22850	MCZ Mammalogy; Harvard MCZ
PRIM	*Colobus*	MCZ:Mamm:47007	MCZ Mammalogy; Harvard MCZ
PRIM	*Colobus*	sbu:rs:81-4	Anatomical Sciences Collection; Stephanie Maiolino
PRIM	*Colobus*	sbu:rs:81-10	Anatomical Sciences Collection; Stephanie Maiolino
PRIM	*Colobus*	sbu:rs:81-14	Anatomical Sciences Collection; Stephanie Maiolino
PRIM	*Colobus*	sbu:rs:83-3	Anatomical Sciences Collection; Stephanie Maiolino
PRIM	*Colobus*	sbu:rs:83-5	Anatomical Sciences Collection; Stephanie Maiolino
PRIM	*Papio*	snrpc:1X4867.W457	Southwest National Primate Research Center; Tim Ryan
PRIM	*Papio*	snrpc:15444.W661	Southwest National Primate Research Center; Tim Ryan
PRIM	*Papio*	snrpc:15283.W666	Southwest National Primate Research Center; Tim Ryan
PRIM	*Papio*	snrpc:1X4248.W689	Southwest National Primate Research Center; Tim Ryan
PRIM	*Papio*	snrpc:1X4851.W670	Southwest National Primate Research Center; Tim Ryan
PRIM	*Papio*	snrpc:15547.W671	Southwest National Primate Research Center; Tim Ryan
PRIM	*Papio*	snrpc:1X4736.W680	Southwest National Primate Research Center; Tim Ryan
PRIM	*Papio*	snrpc:14204.W684	Southwest National Primate Research Center; Tim Ryan
PRIM	*Papio*	snrpc:15539.W686	Southwest National Primate Research Center; Tim Ryan
PRIM	*Papio*	snrpc:1X4779.W692	Southwest National Primate Research Center; Tim Ryan
PRIM	*Papio*	snrpc:15293.W698	Southwest National Primate Research Center; Tim Ryan
PRIM	*Papio*	snrpc:14044.W700	Southwest National Primate Research Center; Tim Ryan
PRIM	*Papio*	snrpc:1X4611.W703	Southwest National Primate Research Center; Tim Ryan
PRIM	*Papio*	snrpc:2X0331.W709	Southwest National Primate Research Center; Tim Ryan
PRIM	*Papio*	snrpc:1X4739.W711	Southwest National Primate Research Center; Tim Ryan
PRIM	*Papio*	snrpc:8895.W717	Southwest National Primate Research Center; Tim Ryan
PRIM	*Papio*	snrpc:15639.W718	Southwest National Primate Research Center; Tim Ryan
PRIM	*Papio*	snrpc:15579.W726	Southwest National Primate Research Center; Tim Ryan

PRIM	*Papio*	snrpc:15140.W729	Southwest National Primate Research Center; Tim Ryan
PRIM	*Papio*	snrpc:8827.W730	Southwest National Primate Research Center; Tim Ryan
PRIM	*Papio*	snrpc:15464.W733	Southwest National Primate Research Center; Tim Ryan
PRIM	*Papio*	snrpc:11639.W734	Southwest National Primate Research Center; Tim Ryan
PRIM	*Papio*	snrpc:8893.W738	Southwest National Primate Research Center; Tim Ryan
PRIM	*Papio*	snrpc:6995.W739	Southwest National Primate Research Center; Tim Ryan
PRIM	*Papio*	snrpc:14167.W745	Southwest National Primate Research Center; Tim Ryan
PRIM	*Papio*	snrpc:1X4194.W753	Southwest National Primate Research Center; Tim Ryan
PRIM	*Papio*	snrpc:16115.W757	Southwest National Primate Research Center; Tim Ryan
PRIM	*Papio*	snrpc:10128.W766	Southwest National Primate Research Center; Tim Ryan
PRIM	*Papio*	snrpc:9417.W777	Southwest National Primate Research Center; Tim Ryan
PRIM	*Papio*	snrpc:7675.W886	Southwest National Primate Research Center; Tim Ryan
PRIM	*Lagothrix*	LAGO:DU:Baa:X	Duke Evolutionary Anthropology Department; Kari Allen
PRIM	*Lagothrix*	LAGO:USNM:MAMM:194342	National Museum of Natural History (SI); NMNH
PRIM	*Lagothrix*	LAGO:USNM:MAMM:269839	National Museum of Natural History (SI); NMNH
PRIM	*Lagothrix*	LAGO:USNM:MAMM:398460	National Museum of Natural History (SI); NMNH
PRIM	*Lagothrix*	LAGO:USNM:MAMM:461930	National Museum of Natural History (SI); NMNH
PRIM	*Lagothrix*	LAGO:USNM:MAMM:461931	National Museum of Natural History (SI); NMNH
PRIM	*Lagothrix*	LAGO:USNM:MAMM:545879	National Museum of Natural History (SI); NMNH
PRIM	*Lagothrix*	LAGO:USNM:MAMM:545881	National Museum of Natural History (SI); NMNH
PRIM	*Lagothrix*	LAGO:USNM:MAMM:545882	National Museum of Natural History (SI); NMNH
PRIM	*Lagothrix*	LAGO:USNM:MAMM:545884	National Museum of Natural History (SI); NMNH
PRIM	*Lagothrix*	LAGO:USNM:MAMM:545886	National Museum of Natural History (SI); NMNH
PRIM	*Lagothrix*	LAGO:USNM:MAMM:545890	National Museum of Natural History (SI); NMNH
PRIM	Callithrix	CAL:AMNH:Mammals:M-200525	AMNH Mammal Collections; AMNH Mammalogy
PRIM	Callithrix	CAL:MCZ:Mamm:37823	MCZ Mammalogy - Harvard MCZ
PRIM	Callithrix	CAL:MCZ:Mamm:BOM-7165	MCZ Mammalogy - Harvard MCZ
PRIM	Callithrix	CAL:USNM:MAMM:503885	National Museum of Natural History (SI); NMNH
PRIM	Lemur	dlc 6276f	Duke Lemur Center Museum of Natural History
PRIM	Lemur	dlc 6808m	Duke Lemur Center Museum of Natural History
PRIM	Lemur	dlc 6848m	Duke Lemur Center Museum of Natural History
PRIM	Lemur	dlc 6862	Duke Lemur Center Museum of Natural History

PRIM	Lemur	dlc 7142f	Duke Lemur Center Museum of Natural History
PRIM	Lemur	MCZ:Mamm:16391	MCZ Mammalogy - Harvard MCZ
PRIM	Lemur	MCZ:Mamm:44903	MCZ Mammalogy - Harvard MCZ

SOM Table 2. Sex and locality distributions for all scored non-human specimens in this study.

Family	Genus	N Surveyed	N Scored	Sex	N Wild	N Zoo	N Other	N Unknown
Hominidae	*Gorilla*	105	67	**3?**	-	1	-	2
				**32F**	29	2	-	1
				**32M**	28	2	-	2
	Pan	98	75	**25?**	12	-	-	13
				**28F**	25	3	-	-
				**22M**	18	2	-	2
	Pongo	106	91	**6?**	1	-	-	5
				**39F**	31	5	-	3
				**46M**	35	10	-	1
Hylobatidae	*Hylobates*	90	75	**3?**	1	1	-	1
				**29F**	27	-	-	2
				**43M**	38	1	-	4
	Symphalangus	21	16	**5F**	2	1	-	2
				**11M**	5	3	-	3
Aotidae	*Aotus*	52	42	**1?**	-	-	-	1
				**26F**	25	1	-	-
				**15M**	15	-	-	-

Atelidae	*Alouatta*	70	59	**8?**	6	-	-	2
				**26F**	21	5	-	-
				**25M**	22	3	-	-
	Ateles	58	49	**5?**	2	2	-	1
				**25F**	25	-	-	-
				**19M**	19	-	-	-
	Lagothrix	64	48	**6?**	2	2	0	2
				**19F**	18	-	-	1
				**23M**	23	-	-	-
Callitrichidae	*Callithrix*	77	59	**7?**	4	2	-	1
				**26F**	15	5	-	6
				**26M**	11	3	-	12
	Leontopithecus	43	40	**12?**	-	6	-	6
				**14F**	-	7	-	7
				**14M**	-	9	-	5
	Saguinus	56	44	**13?**	-	3	-	10
				**10F**	6	2	-	2
				**21M**	11	8	-	2
Cebidae	*Callicebus*	33	30	**20F**	18	2	-	-

				**10M**	6	3	-	1
	Cebus	44	39	**3?**	2	-	-	1
				**12F**	12	-	-	-
				**24M**	24	-	-	-
	Saimiri	35	32	**7?**	3	2		2
				**12F**	12	-	-	-
				**13M**	9	4	-	-
Pitheciidae	*Chiropotes*	38	35	**1?**	1	-	-	-
				**16F**	16	-	-	-
				**18M**	18	-	-	-
	Pithecia	32	30	**2?**	2	-	-	-
				**12F**	12	-	-	-
				**16M**	16	-	-	-
Cercopithecidae	*Cercocebus*	38	27	**7?**	1	5	-	1
				**7F**	5	2	-	-
				**13M**	8	5	-	-
	Cercopithecus	84	64	**16?**	10	6	-	-
				**16F**	11	5	-	-
				**32M**	26	4	-	2

Cercopithecidae	*Chlorocebus*	46	36	**3?**	1	1	-	1
				**16F**	15	1	-	-
				**17M**	13	4	-	-
	Colobus	54	39	**2?**	-	1	-	1
				**25F**	22	2	-	1
				**12M**	10	1	-	1
	Macaca	194	184	**18?**	6	1	-	11
				**63F**	54	-	-	9
				**103M**	96	2	-	5
	Nasalis	43	35	**5?**	2	-	-	3
				**12F**	12	-	-	-
				**18M**	18	-	-	-
	Papio	97	71	**9?**	2	2	-	5
				**27F**	12	13	-	2
				**35M**	25	6	-	4
	Presbytis	37	31	**1?**	1	-	-	-
				**15F**	14	1	-	-
				**15M**	14	1	-	-
	Trachypithecus	53	44	**3?**	1	-	-	2

				**24F**	24	-	-	-
				**17M**	16	1	-	-
Indriidae	*Propithecus*	10	10	**8?**	7	-	-	1
				**1F**	1	-	-	-
				**1M**	1	-	-	-
Lemuridae	*Eulemur*	46	43	**21?**	7	8	-	6
				**11F**	5	4	-	2
				**11M**	5	5	-	1
Lemuridae	*Lemur*	32	30	**8?**	2	4	-	2
				**10F**	1	7	2	-
				**12M**	4	5	3	-
Lemuridae	*Varecia*	26	23	**10?**	6	2	-	2
				**6F**	2	4	-	-
				**7M**	-	7	-	-

		N Wild	N Zoo	N Other	N Unknown
1,782 Surveyed; 1,468 Scored; 671 males (45.71%); 584 females (39.8%); 213 sex unknown (14.51%)	**Total Scored:**	1088	210	5	165
		(74.11%)	(14.30%)	(0.34%)	(11.24%)

SOM Table 3. CBA and % foramen occurrence for human and nonhuman primate genera.

Type	Family	Genus	CBA	%Foramen
APE	Hominidae	*Gorilla*	148	0.432835821
APE	Hominidae	*Pan*	152	0.506666667
APE	Hominidae	*Pongo*	135	0.78021978
APE	Hominidae	*Homo*	111.8	0.122807018
APE	Hylobatidae	*Hylobates*	156.5	0.733333333
APE	Hylobatidae	*Symphalangus*	167	0.9375
PLATYRRHINE	Aotidae	*Aotus*	180	0.023809524
PLATYRRHINE	Atelidae	*Alouatta*	187	0.13559322
PLATYRRHINE	Atelidae	*Ateles*	161.5	0.183673469
PLATYRRHINE	Atelidae	*Lagothrix*	176	0
PLATYRRHINE	Callitrichidae	*Callithrix*	164	0.389830508
PLATYRRHINE	Callitrichidae	*Leontopithecus*	173	0.225
PLATYRRHINE	Callitrichidae	*Saguinus*	170	0.204545455
PLATYRRHINE	Cebidae	*Callicebus*	175	0.033333333
PLATYRRHINE	Cebidae	*Cebus*	172.5	0.051282051
PLATYRRHINE	Cebidae	*Saimiri*	169	0.21875
PLATYRRHINE	Pitheciidae	*Chiropotes*	170	0.171428571
PLATYRRHINE	Pitheciidae	*Pithecia*	182	0.266666667
CATARRHINE	Cercopithecidae	*Cercocebus*	172	0.185185185
CATARRHINE	Cercopithecidae	*Cercopithecus*	174.5	0.21875
CATARRHINE	Cercopithecidae	*Chlorocebus*	171	0.833333333
CATARRHINE	Cercopithecidae	*Colobus*	161	0.384615385
CATARRHINE	Cercopithecidae	*Macaca*	156	0.25
CATARRHINE	Cercopithecidae	*Nasalis*	157	0.314285714

CATARRHINE	Cercopithecidae	*Papio*	154	0.450704225
CATARRHINE	Cercopithecidae	*Presbytis*	154.75	0.709677419
CATARRHINE	Cercopithecidae	*Trachypithecus*	166	0.340909091
STREPSIRRHINE	Indriidae	*Propithecus*	168	0
STREPSIRRHINE	Lemuridae	*Eulemur*	171	0.046511628
STREPSIRRHINE	Lemuridae	*Lemur*	176	0.033333333
STREPSIRRHINE	Lemuridae	*Varecia*	177	0.043478261

SOM Table 4. Juvenile orangutan survey.

**Species**	**Specimen no.**	**Sex**	**Eruption**	**Foramen**
Pongo pygmeaus	ANSP 11810	?	Pre-M1	Pre-dorsum sellae – not scored
Pongo pygmeaus	USNM 520885	F	Pre-M1	Pre-dorsum sellae – not scored
Pongo pygmeaus	USNM 153811	M	M1 erupted; M2 erupting	Pre-dorsum sellae – not scored
Pongo pygmeaus	USNM 302052	F	M1 erupted; M2 erupting	Pre-dorsum sellae – not scored
Pongo pygmeaus	ANSP 12191	?	M1 erupted; M2 erupting	Pre-dorsum sellae – not scored
Pongo abelii	USNM 143586	M	M1 erupted; M2 erupting	YES
Pongo pygmeaus	ANSP 6193	M	M1 erupted; M2 erupting	YES
Pongo pygmeaus	ANSP 13110	F	M1 erupted; M2 erupting	YES
Pongo pygmeaus	USNM 292562	F	M1 erupted; M2 erupting	YES
Pongo abelii	USNM 143595	M	M1 erupted; M2 unerupted	Pre-dorsum sellae – not scored
Pongo abelii	USNM 143599	F	M1 erupted; M2 unerupted	Pre-dorsum sellae – not scored
Pongo pygmeaus	ANSP 12952	?	M1 erupted; M2 unerupted	Pre-dorsum sellae – not scored
Pongo pygmeaus	USNM 396920	F	M1 erupted; M2 unerupted	Pre-dorsum sellae – not scored
Pongo pygmeaus	USNM A49898	?	M1 erupted; M2 unerupted	Pre-dorsum sellae – not scored
Pongo pygmeaus	USNM 197665	M	M1 erupted; M2 unerupted	NO
Pongo pygmeaus	USNM 317197	M	M1 erupted; M2 unerupted	Pre-dorsum sellae – not scored
Pongo pygmeaus	ANSP 12943	?	M1 erupted; M2 unerupted	YES
Pongo pygmeaus	USNM 49435	F	M1 erupted; M2 unerupted	YES
Pongo pygmeaus	USNM 142171	F	M1 erupted; M2 unerupted	YES
Pongo pygmeaus	USNM 283737	F	M1 erupted; M2 unerupted	YES
Pongo pygmeaus	USNM 145322	F	M2 erupted; M3 unerupted	YES
Pongo pygmeaus	USNM 153821	F	M2 erupted; M3 unerupted	YES

SOM Table 5. Power analysis for human and non-human primate samples.

Type[1]	Family	Genus	Sample Size	Observed Proportion	Effect Size	Power
APE	Hominidae	*Homo*	114	0.1228	-0.8547	1
APE	Hominidae	*Gorilla*	67	0.4328	-0.1347	0.1968
APE	Hominidae	*Pan*	75	0.5067	0.0133	0.0515
APE	Hominidae	*Pongo*	91	0.7802	0.5949	0.9999
APE	Hylobatidae	*Hylobates*	75	0.7333	0.4855	0.9876
APE	Hylobatidae	*Symphalangus*	16	0.9375	1.0654	0.9893
PLAT	Aotidae	*Aotus*	42	0.0238	-1.261	1
PLAT	Atelidae	*Alouatta*	59	0.1356	-0.8166	1
PLAT	Atelidae	*Ateles*	49	0.1837	-0.685	0.9977
PLAT	Atelidae	*Lagothrix*	48	0	-1.5708	1
PLAT	Callitrichidae	*Callithrix*	59	0.3898	-0.2222	0.4001
PLAT	Callitrichidae	*Leontopithecus*	40	0.225	-0.5824	0.9576
PLAT	Callitrichidae	*Saguinus*	44	0.2045	-0.6322	0.9872
PLAT	Cebidae	*Callicebus*	30	0.0333	-1.2036	1
PLAT	Cebidae	*Cebus*	39	0.0513	-1.1139	1
PLAT	Cebidae	*Saimiri*	32	0.2188	-0.5974	0.9221

PLAT	Pitheciidae	*Chiropotes*	35	0.1714	-0.717	0.9888
PLAT	Pitheciidae	*Pithecia*	30	0.2667	-0.4855	0.7578
CATA	Cercopithecidae	*Cercocebus*	27	0.1852	-0.6811	0.9428
CATA	Cercopithecidae	*Cercopithecus*	64	0.2188	-0.5974	0.9976
CATA	Cercopithecidae	*Chlorocebus*	36	0.8333	0.7297	0.9922
CATA	Cercopithecidae	*Macaca*	184	0.25	-0.5236	1
CATA	Cercopithecidae	*Papio*	71	0.4507	-0.0988	0.1323
CATA	Cercopithecidae	*Colobus*	39	0.3846	-0.2329	0.3069
CATA	Cercopithecidae	*Nasalis*	35	0.3143	-0.3805	0.6146
CATA	Cercopithecidae	*Presbytis*	31	0.7097	0.4327	0.6734
CATA	Cercopithecidae	*Trachypithecus*	44	0.3409	-0.3238	0.5746
STREP	Lemuridae	*Eulemur*	43	0.0465	-1.136	1
STREP	Lemuridae	*Lemur*	30	0.0333	-1.2036	1
STREP	Lemuridae	*Varecia*	23	0.0435	-1.1507	0.9998
STREP	Indriidae	*Propithecus*	10	0	-1.5708	0.9987

[1]**Type:** APE = greater and lesser apes; PLAT = platyrrhine monkeys; CATA = catarrhine monkeys; STREP = strepsirrhines
